# Proteomics study of primary and recurrent adamantinomatous craniopharyngiomas

**DOI:** 10.1186/s12014-024-09479-4

**Published:** 2024-04-09

**Authors:** Haidong Deng, Ting Lei, Siqi Liu, Wenzhe Hao, Mengqing Hu, Xin Xiang, Ling Ye, Dongting Chen, Yan Li, Fangjun Liu

**Affiliations:** 1 Department of Neurosurgery, Sanbo Brain Hospital, Capital Medical University, Beijing, 100093 China; 2 Beijing Key Laboratory of New Drug Mechanisms and Pharmacological Evaluation Study, Department of Pharmacology, Institute of Materia Medica, Chinese Academy of Medical Sciences and Peking Union Medical College, Beijing, 100050 China; 3 School of Basic Medical Sciences, Tianjin Medical University, Tianjin, 300070 China

**Keywords:** Craniopharyngioma, adamantinomatous type, Immune microenvironment, Proteomics, Cathepsin K, Epithelial tumour, Differential expression

## Abstract

**Background:**

Adamantinomatous craniopharyngiomas (ACPs) are rare benign epithelial tumours with high recurrence and poor prognosis. Biological differences between recurrent and primary ACPs that may be associated with disease recurrence and treatment have yet to be evaluated at the proteomic level. In this study, we aimed to determine the proteomic profiles of paired recurrent and primary ACP, gain biological insight into ACP recurrence, and identify potential targets for ACP treatment.

**Method:**

Patients with ACP (*n* = 15) or Rathke’s cleft cyst (RCC; *n* = 7) who underwent surgery at Sanbo Brain Hospital, Capital Medical University, Beijing, China and received pathological confirmation of ACP or RCC were enrolled in this study. We conducted a proteomic analysis to investigate the characteristics of primary ACP, paired recurrent ACP, and RCC. Western blotting was used to validate our proteomic results and assess the expression of key tumour-associated proteins in recurrent and primary ACPs. Flow cytometry was performed to evaluate the exhaustion of tumour-infiltrating lymphocytes (TILs) in primary and recurrent ACP tissue samples. Immunohistochemical staining for CD3 and PD-L1 was conducted to determine differences in T-cell infiltration and the expression of immunosuppressive molecules between paired primary and recurrent ACP samples.

**Results:**

The bioinformatics analysis showed that proteins differentially expressed between recurrent and primary ACPs were significantly associated with extracellular matrix organisation and interleukin signalling. Cathepsin K, which was upregulated in recurrent ACP compared with that in primary ACP, may play a role in ACP recurrence. High infiltration of T cells and exhaustion of TILs were revealed by the flow cytometry analysis of ACP.

**Conclusions:**

This study provides a preliminary description of the proteomic differences between primary ACP, recurrent ACP, and RCC. Our findings serve as a resource for craniopharyngioma researchers and may ultimately expand existing knowledge of recurrent ACP and benefit clinical practice.

**Supplementary Information:**

The online version contains supplementary material available at 10.1186/s12014-024-09479-4.

## Background

The annual incidence of craniopharyngioma is approximately 0.5–2.5 per 1 million individuals. The disease has two incidence peaks, occurring at 5–14 and 50–75 years [1, 2]. Craniopharyngioma is generally believed to arise from the remnants of Rathke’s pouch [2]. Another less common lesion of the sellar and parasellar regions is Rathke’s cleft cyst (RCC), which is also considered to arise from Rathke’s pouch [3]. Nuclear accumulation of β-catenin is the hallmark of adamantinomatous craniopharyngioma, while BRAF mutation is the hallmark of papillary craniopharyngioma [4]. RCC shows none of the above characteristics [5]. Previous studies have reported the transitional state between RCC and craniopharyngioma [6–8].Nearly all cases of craniopharyngioma in children and adolescents are of the adamantinomatous type (ACP), which are cystic-solid tumours [2]. ACP profoundly affects the hypothalamic–pituitary system, with the tumour and required surgeries leading to hypothalamic destruction. Consequently, the neuroendocrine function is severely compromised, particularly in children, leading to growth and developmental disorders, infertility, polydipsia, and polyuria. These symptoms become more pronounced in patients with tumour recurrence, with a recurrence rate as high as 26% [9]. Therefore, extensive research on ACP is essential to develop effective drugs that prevent recurrence [10].

ACP has a distinct capsule whose inner surface is lined with palisading epithelial cells, and the cyst fluid is enriched with inflammatory factors such as TNF-α, IFN-γ, IL-1β, and IL-6. Qi et al. observed that the palisading epithelial cells of ACP exhibit significantly high expression of vimentin and significantly decreased expression of CDH1 and β-catenin. This suggests that epithelial–mesenchymal transition (EMT) plays a crucial role in ACP progression [11]. Inflammatory factors induce and promote EMT in epithelial cells [12]. Furthermore, the abnormal upregulation of vimentin and downregulation of CDH1 have been associated with tumour recurrence and impaired hypothalamic function following surgical intervention in patients with ACP [13]. These findings support the potential involvement of EMT in tumour migration and recurrence.

Proteomics is a powerful tool for characterising molecular diseases and identifying potential cancer treatment targets. However, our understanding of the molecular features of recurrent and primary ACP at the proteomic level is limited. Therefore, this study aimed to determine the proteomic profiles of paired recurrent and primary ACPs, gain biological insight into ACP recurrence, and identify potential targets for ACP treatment.

## Methods

### Study sample

Patients with ACP or RCC who underwent surgery at Sanbo Brain Hospital, Capital Medical University, Beijing, China and received pathological confirmation of ACP or RCC were enrolled in this study. None of the participants received radiotherapy, chemotherapy, or any other form of antitumour therapy before surgery. Informed consent was obtained from all participants or their parents or legal guardians. The study was approved by the Institutional Review Board of Sanbo Brain Hospital (approval nos. SBNK-YJ-2022-010-01, SBNK-YJ-2023-006-01). All methods were performed in accordance with the guidelines of the Institutional Review Board of Sanbo Brain Hospital and the Declaration of Helsinki. All patients’ information can be found in supplementary table S1.

### Formalin-fixed and paraffin-embedded sample preparation

Add 20 µL of extraction solution (containing 10mM Tris, 0.8mM EDTA, and 0.2% Zwittergent 3–16) on the section, scrape off the paraffin-embedded tissue with a knife and collect it directly into a 1.5 mL centrifuge tube. The FFPE sample was deparaffinized at 100 °C for 20 min. Then, the sample was reduced with 20 mM dithiothreitol (DTT) for 2 h at 60 °C with sonication, alkylated with 55 mM iodoacetamide (IAM) for 45 min at room temperature (24–27 °C) in the dark, and loaded onto a 30 kD filter, and centrifuged at 14,000 g for 20 min. The protein samples on the filter were washed five times with 20 mM Tris solution. The protein samples on the filter were digested with trypsin (1:50) at 37 °C overnight, and the peptides were collected by centrifugation after enzymatic digestion.

### Liquid chromatography with tandem mass spectrometry

Orbitrap Exploris 480 (Thermo Scientific, USA) coupled with the Ultimate 3000 (Thermo Scientific, USA) was used for analysis in the data-independent acquisition-mass spectrometry (DIA-MS) mode. The digested peptides were separated on an RP monolithic capillary LC column (75 μm×500 mm; Uritech, Beijing, China). The eluted gradient was 5–30% buffer B2 (0.1% formic acid, 99.9% acetonitrile; flow rate: 1.5 µL/min), and peptides were eluted for 25 min.

For DIA analysis, a variable isolation window with 60 windows was employed for MS acquisition. According to the precursor m/z distribution of the pooled sample, the precursor ion number was equalized in each isolation window. The full scan range was set from 350 to 1200 m/z and screened at a resolution of 120,000, followed by DIA scans with a resolution of 30,000 (higher-energy C-trap dissociation [HCD] collision energy: 30%; AGC target: 200%; maximum injection time: 50 ms).

### Data processing

The raw DIA data were analyzed by Spectronaut Pulsar 17.1 (Biognosys, Zurich, Switzerland) with default settings. In brief, the retention time prediction type was set to dynamic iRT. Interference correction on the MS2 level was enabled. Peptide intensity was calculated by summing the peak areas of their respective fragment ions for MS2, and the protein intensity was calculated by summing the intensity of their respective peptides. Cross-run normalization was enabled to correct for systematic variance in the LC-MS/MS performance, and a local normalization strategy was used. The normalization was based on the assumption that on average, a similar number of peptides was up-regulated and down-regulated, and the majority of the peptides within the sample were not regulated across runs or during the retention time. Protein inference was performed with the ID picker algorithm implemented in Spectronaut. All results were filtered by a Q value cutoff of 0.01 (corresponding to an FDR of 1%).

### Differentially expressed proteins

Differential protein expression levels among primary ACP, recurrent ACP, and RCC were estimated using a two-tailed t-test with Microsoft Office Excel 2019 (Microsoft Corp, Redmond, WA, USA). Proteins were considered differentially expressed if the *P*-value was < 0.05 and the fold change was > 2.

### Bioinformatics analyses

Analyses of differentially expressed proteins (DEPs) in primary ACP, recurrent ACP, and RCC were performed using the Metascape bioinformatics tool (https://metascape.org/gp/index.html#/main/step1, v3.5.20230501) [14], wherein R/P represents recurrent versus primary ACP, P/RCC represents primary ACP versus RCC, and R/RCC represents recurrent ACP versus RCC. Pathway and process enrichment analyses have been carried out with default settings.

### Hub node clustering

Protein–protein interaction (PPI) networks of R/P DEPs were predicted using STRING (Search Tool for the Retrieval of Interacting Genes/Proteins, https://cn.string-db.org/, version 11.5), a web resource from the ELIXIR infrastructure for PPI networks and functional enrichment analysis [15] that includes direct (physical) and indirect (functional) interactions. The minimum required interaction score was set as 0.400.

PPI clustering was conducted using the cytoHubba application (version 0.1), which uses 12 different algorithms to predict hub nodes, installed in Cytoscape (version 3.9.1) [16], an open-source software platform utilized for visualising interaction networks and integrating them with various algorithms. Proteins were scored 1 point each time they appeared in the top 10% of any algorithm. Thereafter, proteins with > 4 points were selected and screened using the condition ‘R/P > 2.0 and *P* < 0.01’.

### Ingenuity pathway analysis pathway and network analysis

The list of DEPs identified in the formalin-fixed, paraffin-embedded samples was uploaded to the Ingenuity Pathway Analysis (IPA) software (Ingenuity Systems, Mountain View, CA, USA). The biological processes and canonical pathway analysis functions included in the software were used to interpret the differentially expressed data. Bubble charts were created with Sangerbox 3.0 (http://www.sangerbox.com/tool) [17].

### Flow cytometry

Freshly resected tumour tissues were washed with cold 1× phosphate-buffered saline (PBS), cut into small pieces, and digested using the Human Tumour Dissociation Kit (130-095-929; Miltenyi biotech, Auburn, CA, USA) according to the manufacturer’s instructions. Briefly, tumour tissues were digested using 2.2 mL of Roswell Park Memorial Institute 1640 medium (supplemented with 100 µL of enzyme H, 10 µL of enzyme R, and 12.5 µL of enzyme A) at 37 °C for 1 h. Subsequently, 10 mL of cold 1× PBS was added to stop enzymatic digestion, and the cell suspension was filtered through a 40 μm cell strainer and centrifuged at 300 g for 7 min. After complete aspiration of the supernatant, the cells were washed thrice with cold 1× PBS, centrifuged at 300 g for 5 min, and resuspended in cell-staining buffer (420,201; BioLegend, San Diego, CA, USA). The cells were stained using the following fluorophore-conjugated monoclonal antibody panel: APC-Cy7 anti-human CD45 (368,515; BioLegend, San Diego, CA, USA), BV421 anti-human CD3 (317,343; BioLegend, San Diego, CA, USA), PE-anti-human CD4 (300,507; BioLegend, San Diego, CA, USA), FITC anti-human CD8 (344,703; BioLegend, San Diego, CA, USA), and APC anti-human PD-1 (329,907; BioLegend, San Diego, CA, USA). Fluorescence-minus-one (FMO) controls and the experimental samples were stained for 20 min in the dark on ice. The samples were then centrifuged at 200 g for 5 min at 4 °C, and the cells were resuspended in 300 µL of cell-staining buffer. The cells were analysed using the CytoFLEX S flow cytometer (Beckman Coulter, Brea, CA, USA) and the CytExpert software (Beckman Coulter, Brea, CA, USA). For exhausted T cells, gates were drawn based on the FMO controls.

### Western blotting

Tumour tissues were collected from patients with primary ACP and recurrent ACP. Proteins were extracted from the tissues using a tissue protein extraction kit (CW0891; Cwbio, Nanjing, China). Lysates containing 10 µg of protein were loaded onto a sodium dodecyl–sulphate polyacrylamide gel and subsequently blotted onto a polyvinylidene difluoride membrane. After blocking for 1 h using 5% skim milk (W/V, 1× TBST [0.2% Tween-20 in Tris-buffered saline]) at room temperature (24–27 °C), the membrane was incubated overnight at 4 °C with one of the following antibodies: anti-β-actin (8457; Cell Signaling Technology, Danvers, MA, USA), anti-GAPDH (A19056; Abclonal, Wuhan, China), anti-LUM (ab168348; Abcam, Cambridge, UK), anti-CTSK (ab187647; Abcam, Cambridge, UK), anti-MMP-9 (ab38898; Abcam, Cambridge, UK), anti-VEGF (ab69479; Abcam, Cambridge, UK), anti-CDH2 (13,117; Cell Signaling Technology, Danvers, MA, USA), anti-CDH1 (3195; Cell Signaling Technology, Danvers, MA, USA), anti-MMP-2 (87,809; Cell Signaling Technology, Danvers, MA, USA) and anti-IDO-1 (ab211017; Abcam, Cambridge, UK). Following three washes with TBST, the membrane was incubated with horseradish peroxidase (HRP)-conjugated anti-mouse (ZB-2305; ZSGB-BIO, Beijing, China) or anti-rabbit secondary antibody (ZB-2301; ZSGB-BIO, Beijing, China) for 1 h at room temperature (24–27 °C) and then washed three more times with TBST. Finally, the proteins on the membrane were measured using the Invitrogen iBright FL1000 Imaging System (Thermo Fisher, Waltham, MA, USA) with Super ECL Plus hypersensitive chemiluminescence solution (P1050; Applygen Technologies Inc., Beijing, China).

### IHC

The tumour tissues were fixed in 10% neutral buffered formalin and embedded in paraffin. Thereafter, the tumour sections were deparaffinised and rehydrated. After antigen retrieval, the sections were stained with anti-PD-L1 (ab205921; Abcam, Cambridge, UK) or anti-CD3 (ZA-0503; ZSGB‑BIO, Beijing, China)) antibody overnight at 4 °C and HRP-conjugated anti-rabbit secondary antibody (ZB-2301; ZSGB-BIO, Beijing, China) at 37 °C for 1 h. CD3 and PD-L1 expression was visualised using diaminobenzidine staining.

## Results

### Patient selection and clinical characteristics

**Fig. 1:**
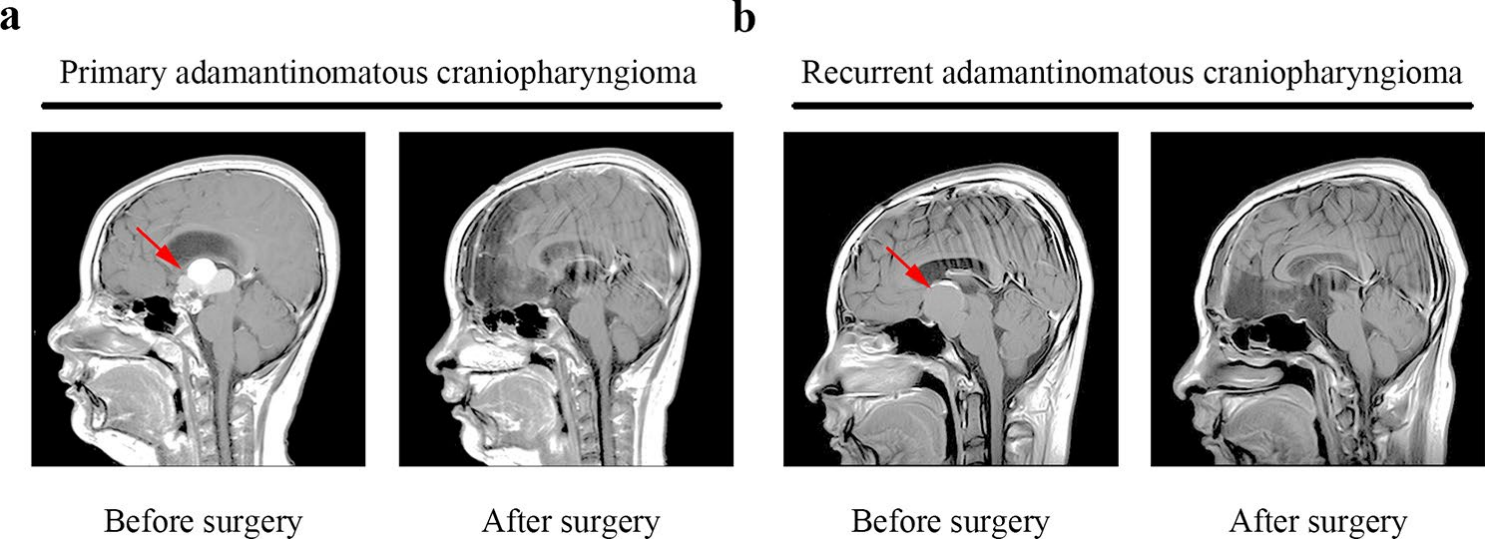
Representative magnetic resonance imaging of primary (**a**) and recurrent (**b**) adamantinomatous craniopharyngioma before and after surgery. Red arrows indicate tumour lesions

A total of 15 patients diagnosed with ACP and 7 patients diagnosed with RCC were enrolled in this study. In proteomic study, seven matched patients with primary and recurrent ACPs were studied (Table 1). Representative magnetic resonance images for these patients are shown in Fig. 1.

**Table 1:** Clinical characteristics of patients with adamantinomatous craniopharyngioma in proteomic studies

Variables	Patients (n = 7)
Sex (female : male)	3:4
Median age at primary ACP diagnosis (years)	5
Median age at recurrent ACP diagnosis (years)	10
Mean time to recurrence (months)	44

ACP, adamantinomatous craniopharyngioma

### Proteomic differences between recurrent ACP, primary ACP, and RCC

**Fig. 2:**
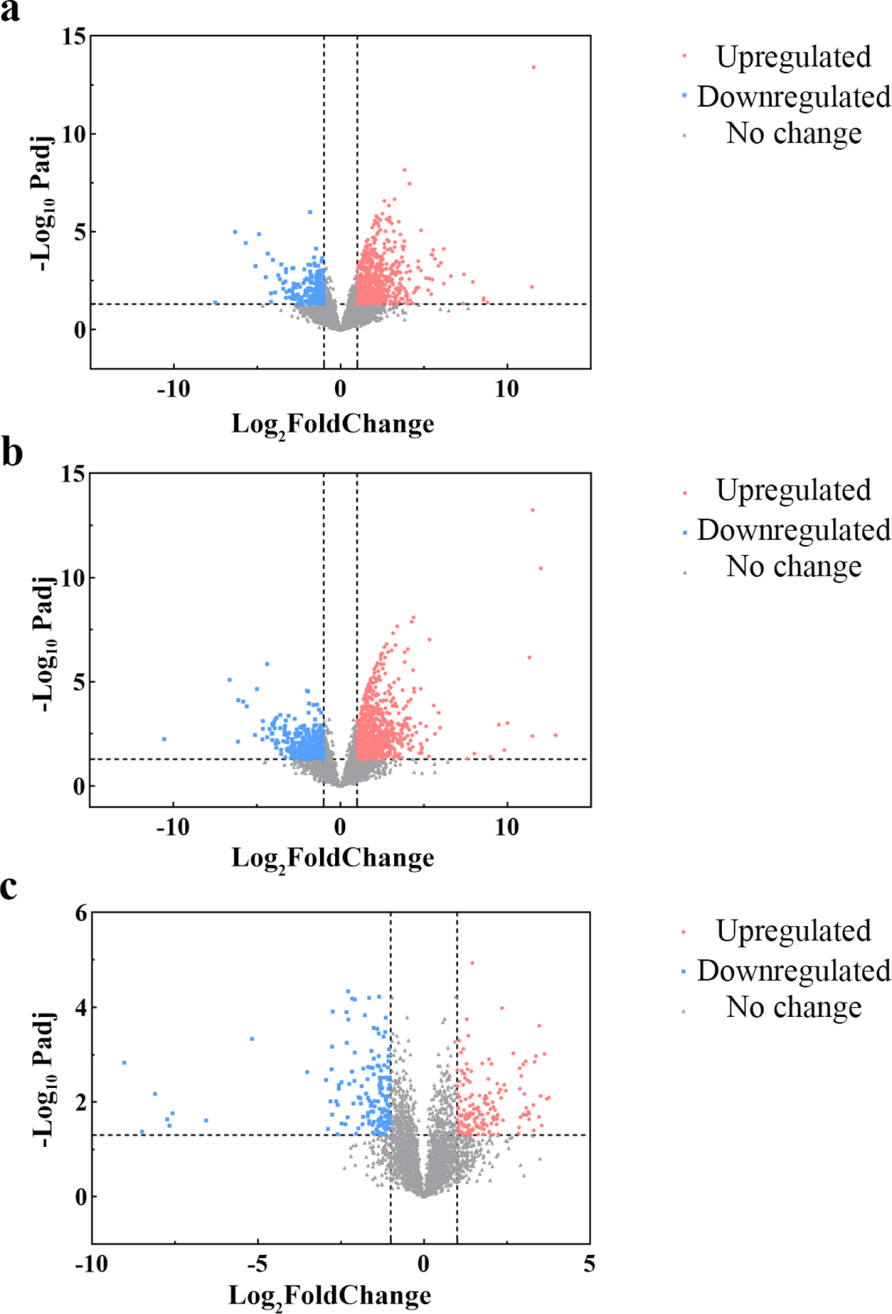
Volcano plot of differentially expressed proteins. (**a**) Volcano plot of 1362 differentially expressed proteins between primary adamantinomatous craniopharyngioma and Rathke’s cleft cyst. (**b**) Volcano plot of 1017 differentially expressed proteins between recurrent adamantinomatous craniopharyngioma and Rathke’s cleft cyst. (**c**) Volcano plot of 317 differentially expressed proteins between recurrent and primary adamantinomatous craniopharyngiomas

In total, 1362 DEPs (997 upregulated and 365 downregulated proteins) were found between primary ACP and RCC (Fig. 2a). The top 10 upregulated proteins were ENPP4, FAM169A, SLC4A4, ROBO1, SQOR, IGF2BP2, OAS3, COMMD10, WNT6, and EMILIN1, whereas the top 10 downregulated proteins were IGHV2-70D, IGHV1-18, IGHV3-72, IGLV9-49, IGHV3-74, IGHV3-73, IGHV3-49, IGHV1-45, IGLV3-9, and NUDT4B.

We identified 1017 DEPs (798 upregulated and 219 downregulated proteins) between recurrent ACP and RCC (Fig. 2b). The top 10 upregulated proteins were DUSP12, PGAM2, SLTM, FYCO1, DNAAF5, DNTTIP2, KRTAP3-2, INTS1, KRTAP4-4, and KRTAP20-2, and the top 10 downregulated proteins were ODF3B, COLEC12, WFDC2, SPATA7, SPATA18, CREG1, AGR3, BPIFA1, GSTA5, and CROCC2.

We identified 317 DEPs (165 upregulated and 152 downregulated proteins) between recurrent and primary ACPs (Fig. 2c). The top 10 upregulated proteins were LUM, OGN, KRTAP7-1, OGT, PRELP, ADAMDEC1, DCN, CXCL5, CTSK, and AMPD3, whereas the top 10 downregulated proteins were CCND2, MEGF6, LHB, BPIFA1, EPHB4, NLGN4X, U2SURP, PPP1R12B, TOR1AIP2, and ARHGEF16.

**Fig. 3:**
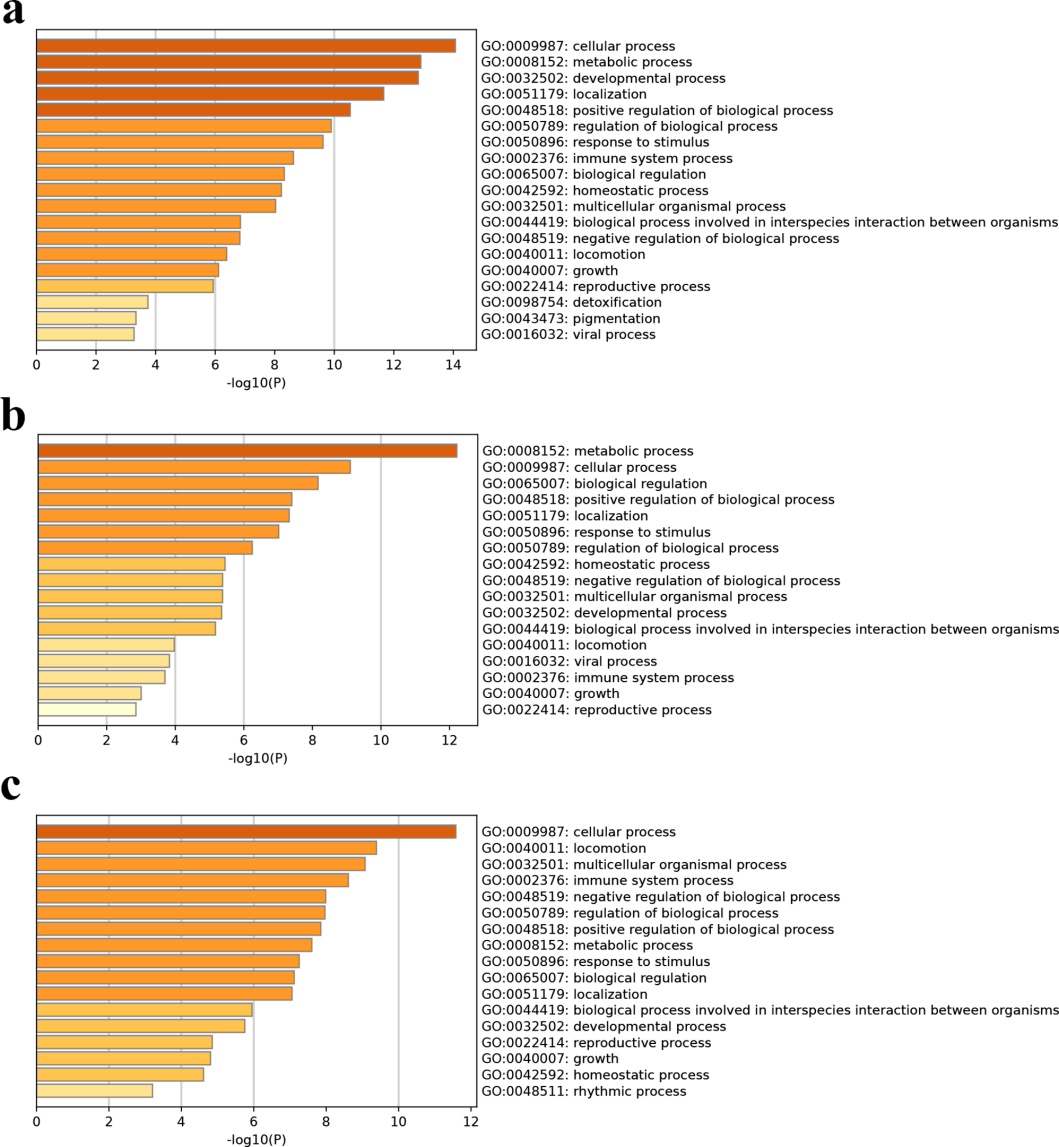
Gene Ontology (GO) enrichment analysis of differentially expressed proteins (DEPs) using Metascape. GO enrichment analysis of DEPs between (**a**) primary adamantinomatous craniopharyngioma (ACP) and Rathke’s cleft cyst, (**b**) recurrent ACP and Rathke’s cleft cyst, and (**c**) primary and recurrent ACPs.

Enrichment analysis of the DEPs was performed to identify common cellular processes and pathways among the three pairs of conditions. Gene Ontology analysis of the P/RCC DEPs revealed enrichment of cellular, metabolic, and developmental processes, localisation, and positive regulation of biological processes (Fig. 3a). The R/RCC DEPs were associated with metabolic and cellular processes, biological regulation, positive regulation of biological processes, localisation, response to stimuli, and regulation of biological processes (Fig. 3b). Finally, the R/P DEPs were associated with cellular processes, locomotion, multicellular organismal processes, immune system processes, negative and positive regulation of biological processes, metabolic processes, response to stimuli, biological regulation, and localisation (Fig. 3c).

**Fig. 4:**
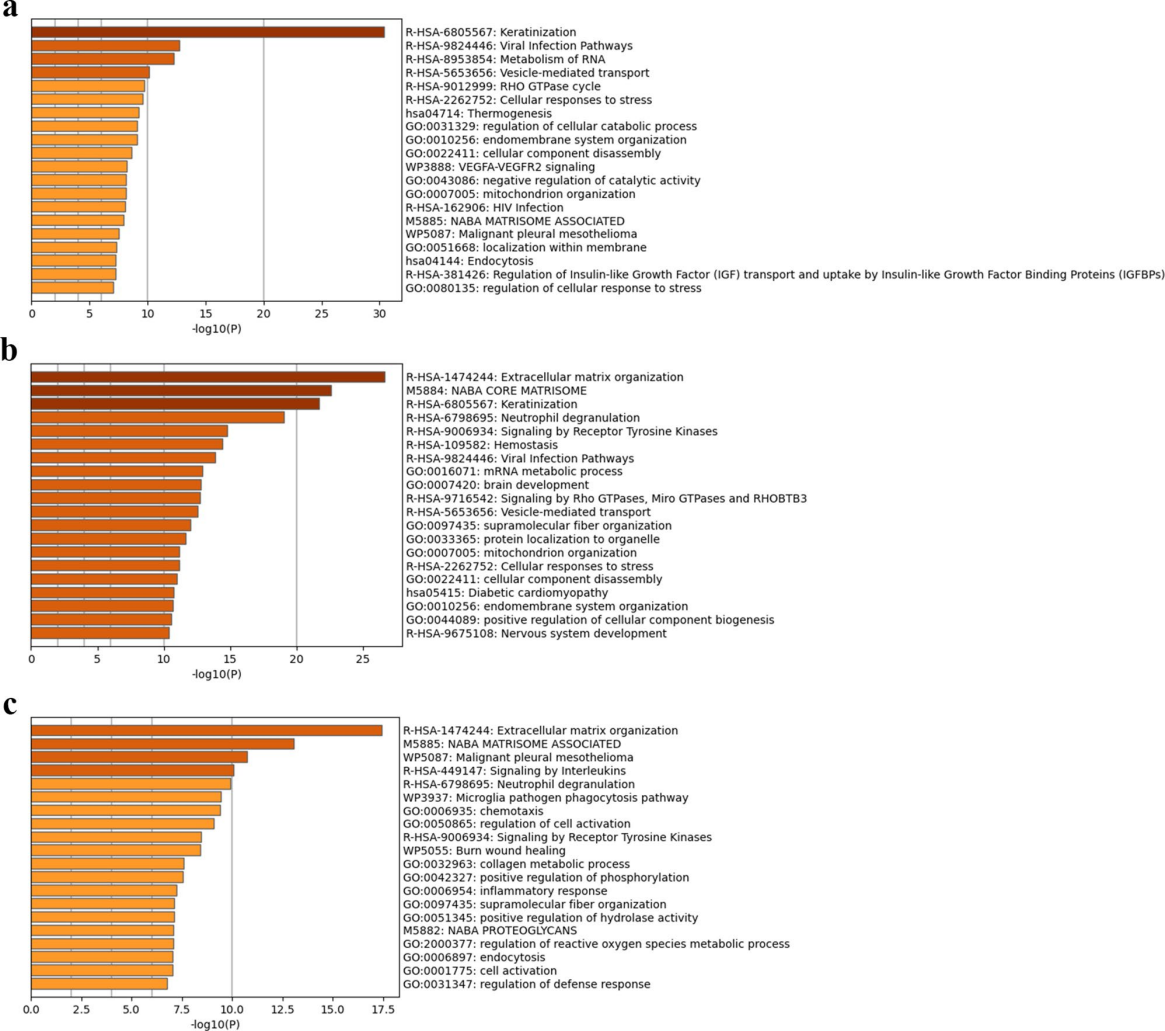
Top 20 enriched ontology clusters of differentially expressed proteins (DEPs) analysed using Metascape. Top 20 enriched DEP clusters between (**a**) primary adamantinomatous craniopharyngioma (ACP) and Rathke’s cleft cyst, (**b**) recurrent ACP and Rathke’s cleft cyst, and (**c**) primary and recurrent ACPs.

The top 20 enriched pathways for each pair of conditions are shown in Fig. 4. The P/RCC DEPs were associated with keratinisation, viral infection pathways, RNA metabolism, and vesicle-mediated transport (Fig. 4a). The R/RCC DEPs were associated with extracellular matrix (ECM) organisation, Naba core matrisome, keratinisation, and neutrophil degranulation (Fig. 4b). Further, the R/P DEPs were enriched for ECM organisation, Naba matrisome-associated pathway, malignant pleural mesothelioma, and interleukin signalling (Fig. 4c).

To further investigate the aetiology of recurrent ACP, hub nodes were identified on the basis of the PPI clustering results (Fig. S1) and filter conditions. Ultimately, LUM, CTSK, COL14A1, and CXCL12 were identified as hub nodes in recurrent ACP but not in primary ACP (Table 2). Interestingly, all four proteins were expressed at higher levels in the patients with recurrent ACP than in those with primary ACP.

Notably, we investigated the upregulated and downregulated R/P DEPs in each patient and determined the overlap to characterise the generalisability of the findings. CTSK was upregulated in all the paired samples; however, no protein was commonly downregulated.

**Table 2:** Hub nodes clustered using cytoHubba and filtered

Hub nodes	Gene symbol	R/P	P
Lumican	*LUM*	3.771223	0.008054
Cathepsin K	*CTSK*	3.339937	0.001418
Collagen type XIV alpha 1 chain	*COL14A1*	3.025944	0.006751
C-X-C motif chemokine ligand 12	*CXCL12*	2.935904	0.002831

R/P: Recurrent adamantinomatous craniopharyngioma versus primary adamantinomatous craniopharyngioma

### IPA pathway analysis

**Fig. 5:**
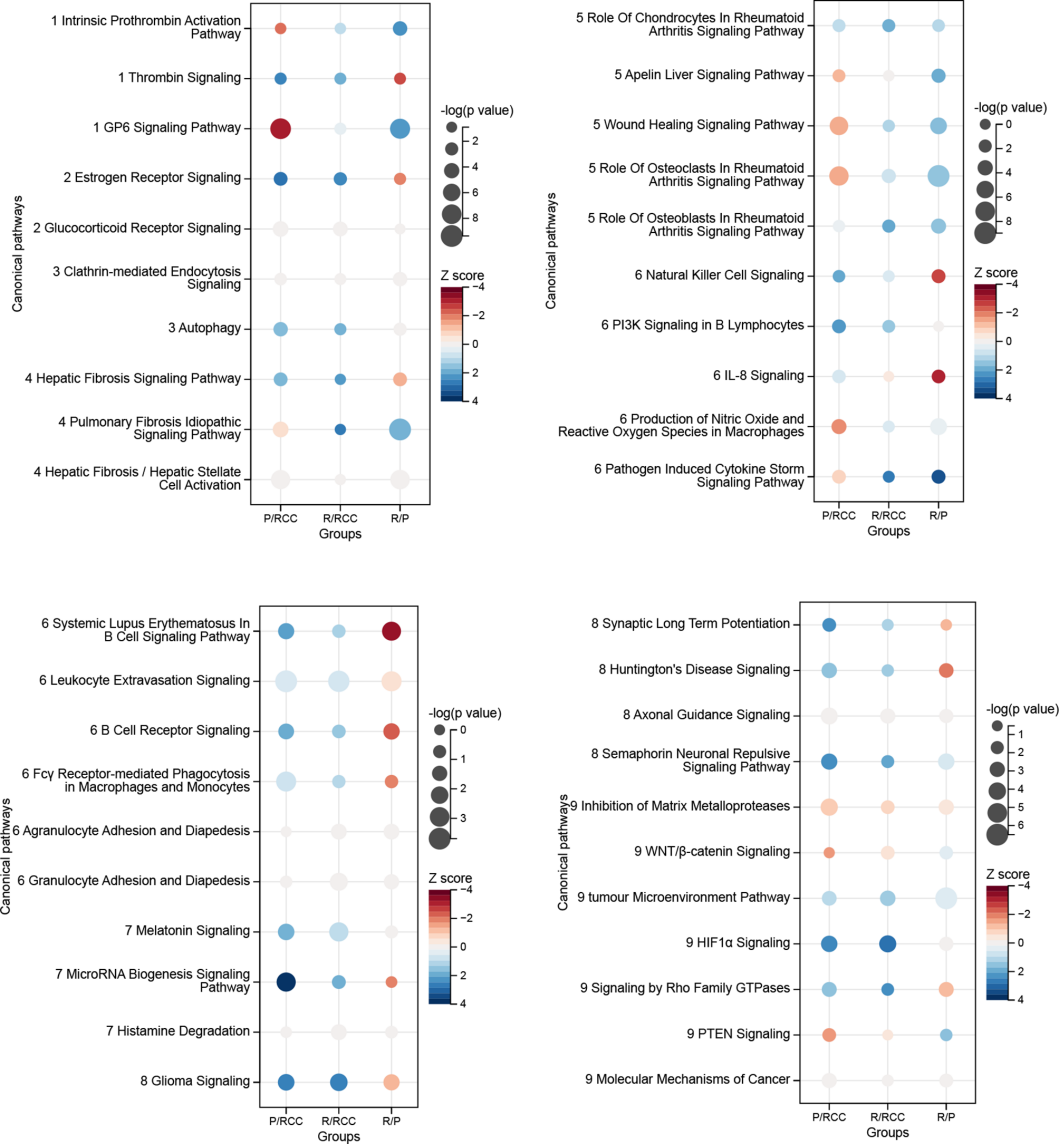
Canonical pathway analysis of differentially expressed proteins between Rathke’s cleft cyst (RCC), primary adamantinomatous craniopharyngioma (ACP), and recurrent ACP. P/RCC, primary ACP versus RCC; R/RCC, recurrent ACP versus RCC; R/P, recurrent ACP versus primary ACP. 1, coagulation; 2, endocrine; 3, endocytosis; 4, fibrosis; 5, other; 6, immune; 7, metabolism; 8, neuron; 9, tumour related

Canonical pathway analysis using IPA indicated that primary and recurrent ACPs involve intricate regulation of coagulation, endocrine function, endocytosis, fibrosis, immunity, metabolism, neuronal activity, tumour-related activity, and other functions (Fig. 5). Additionally, some pathways were inversely regulated in primary and recurrent ACPs compared with those in RCC. Thus, recurrent ACP may involve diverse coagulation, fibrosis, and immunity-related mechanisms. The P/RCC DEPs were associated with activated thrombin signaling, activated estrogen receptor signaling, activated natural killer cell signaling, activated PI3K signaling in B lymphocytes, activated systemic lupus erythematosus in B cell signaling pathway, activated microRNA biogenesis signaling pathway, activated glioma signaling, activated synaptic long term potentiation, activated Huntington’s disease signaling, activated axonal guidance signaling, activated semaphorin neuronal repulsive signaling pathway, activated inhibition of matrix metalloproteases, activated tumour microenvironment pathway, activated HIF1-α signaling, activated Signaling by Rho family GTPases, activated PTEN signaling, activated molecular mechanisms of cancer, inhibited intrinsic prothrombin activation pathway and inhibited GP6 signaling pathway. The R/RCC DEPs were associated with activated hepatic fibrosis signaling pathway, activated pulmonary fibrosis idiopathic signaling pathway, activated role of osteoblasts in rheumatoid arthritis signaling pathway, activated pathogen induced cytokine storm signaling pathway, activated glioma signaling, activated molecular mechanisms of cancer, activated axonal guidance signaling, activated inhibition of matrix metalloproteases, activated WNT/β-catenin signaling, activated tumour microenvironment pathway and activated HIF1-α signaling. The R/P DEPs were associated with activated intrinsic prothrombin activation pathway, activated GP6 signaling pathway, inhibited thrombin signaling, inhibited natural killer cell signaling, inhibited IL-8 signaling, inhibited systemic lupus erythematosus in B cell signaling pathway, inhibited B cell receptor signaling and inhibited Huntington’s disease signaling.

### Flow cytometry

**Fig. 6:**
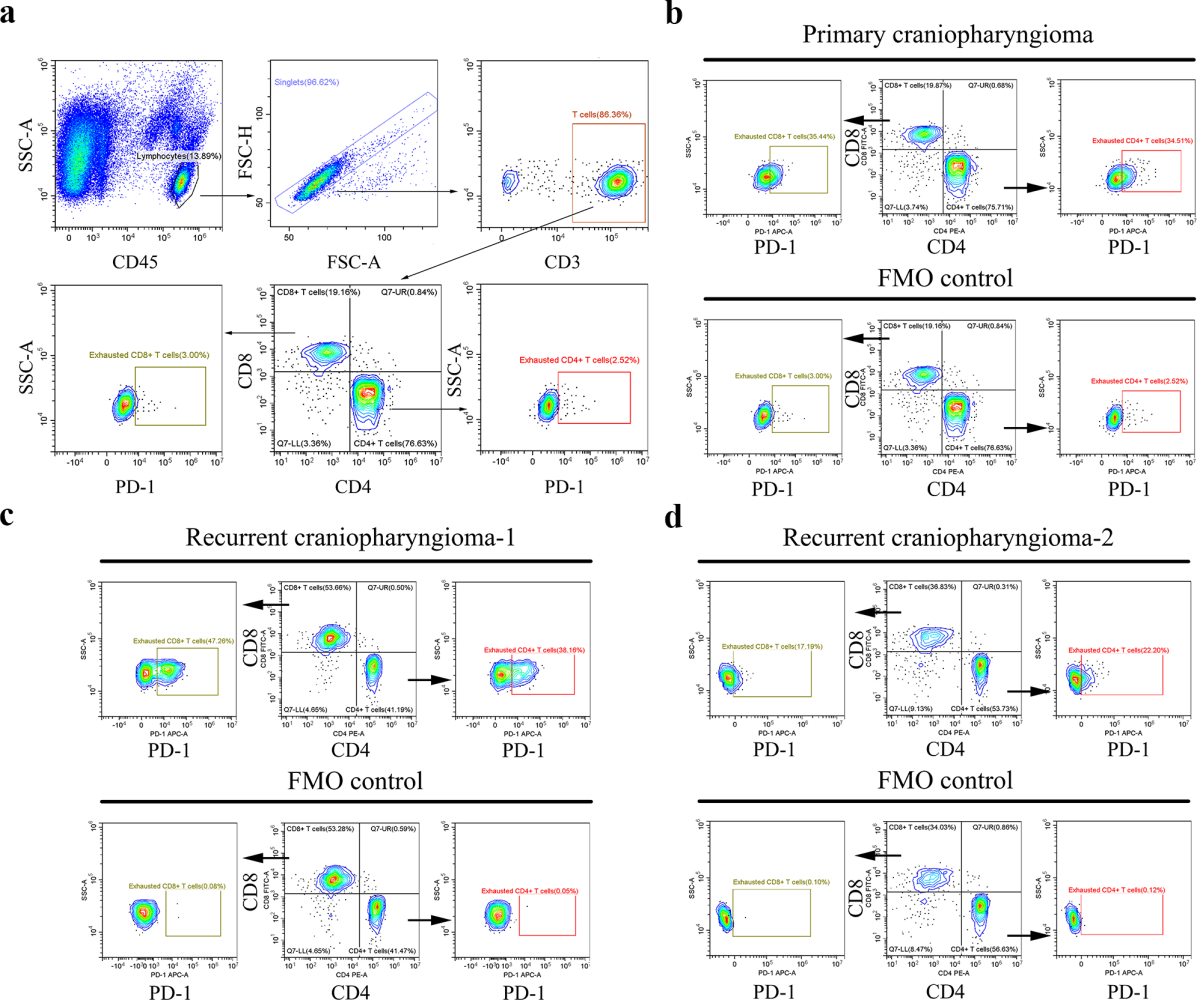
Flow cytometry analysis of tumour-infiltrating lymphocytes in primary and recurrent adamantinomatous craniopharyngioma (ACP). (**a**) gating strategies for analysis of tumour-infiltrating lymphocytes. (**b**) Tumour-infiltrating T cell exhaustion in primary ACP patient. (**c**) Tumour-infiltrating T cell exhaustion in recurrent ACP patient-1. (**d**) Tumour-infiltrating T cell exhaustion in recurrent ACP patient-2. ACP, adamantinomatous craniopharyngioma; SSC-A, side-scatter area; FSC-A, forward-scatter area; FSC-H, forward-scatter height; FMO, fluorescence-minus-one

The gating strategies used in the flow cytometry analysis are shown in Fig. 6a. Lymphocytes were gated according to the side-scatter area and CD45; singlets, the forward-scatter area and height; and T cells, CD3 expression. CD4 + and CD8 + T cells were gated according to their specific markers. Subsequently, exhausted T cells were gated according to PD-1 expression using the FMO controls. Three samples (two recurrent and one primary ACP) were assessed, all of which were rich in lymphocytic infiltration. For recurrent ACP-1, 53.66% of the T cells were CD8 + and 41.19% were CD4 + T cells. Among them, 47.26% of the CD8 + T cells and 38.16% of the CD4 + T cells were exhausted, with high PD-1 expression (Fig. 6c). For recurrent ACP-2, 36.83% of the T cells were CD8 + and 53.73% were CD4 + T cells; 17.19% and 22.20% of the CD8 + and CD4 + T cells, respectively, were exhausted (Fig. 6d). For primary ACP, 19.87% of the T cells were CD8 + and 75.71% were CD4 + T cells; among them, 35.44% of the CD8 + and 35.53% of the CD4 + T cells were exhausted (Fig. 6b).

### Western blotting

**Fig. 7:**
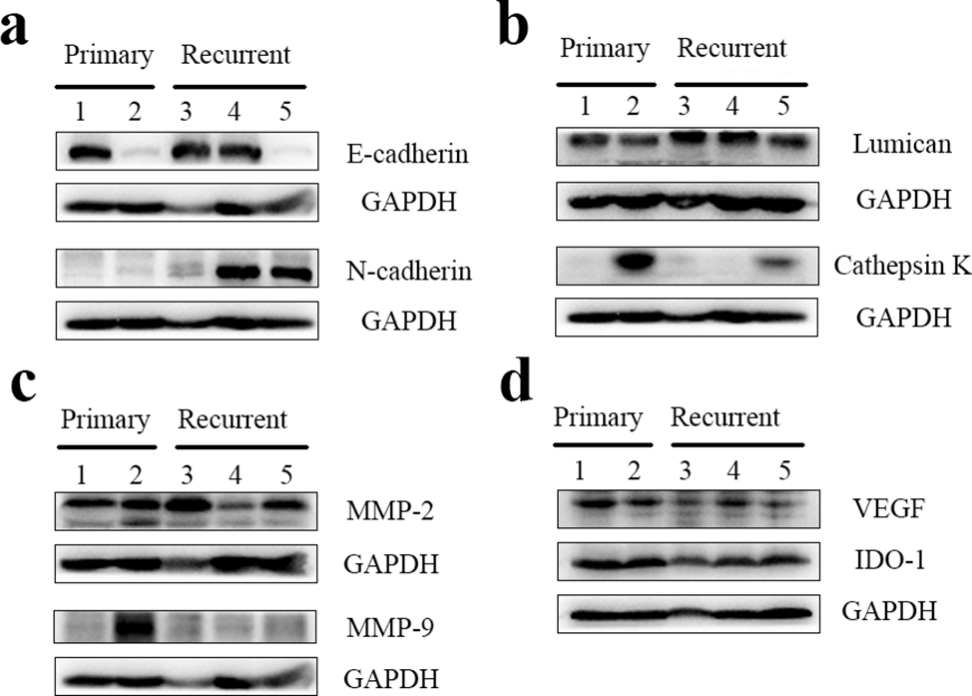
Western blotting analysis of primary and recurrent adamantinomatous craniopharyngioma (ACP) tissues. (**a**) Epithelial- mesenchymal transition associated proteins. (**b**) Key proteins identified in proteomic study. (**c**) Matrix metalloproteinases. (**d**) Key protein in tumour angiogenesis and immune tolerance. Lanes 1 and 2: primary ACP; lanes 3, 4, and 5: recurrent ACP.

Western blotting had been conducted using tumour tissues from two patients with primary ACP and three with recurrent ACP. The analysis revealed that CDH2 expression was higher in recurrent ACP than in primary ACP (Fig. 7a). Tumour tissues from patients 1, 3, and 4 showed high CDH1 expression, whereas those from patients 2 and 5 showed decreased CDH1 expression. No difference in MMP-2 expression was observed between primary and recurrent ACPs. Patient 2, who was diagnosed with recurrent ACP after primary tumour resection, showed the highest expression levels of CTSK (Fig. 7b) and MMP-9 (Fig. 7c). Although the proteomic data suggested upregulated LUM expression in recurrent ACP tissues, this was not observed in the western blotting analysis (Fig. 7b). In addition, on exploring the expression of VEGF and IDO-1, we found no significant differences between primary and recurrent ACPs (Fig. 7d).

### IHC

**Fig. 8:**
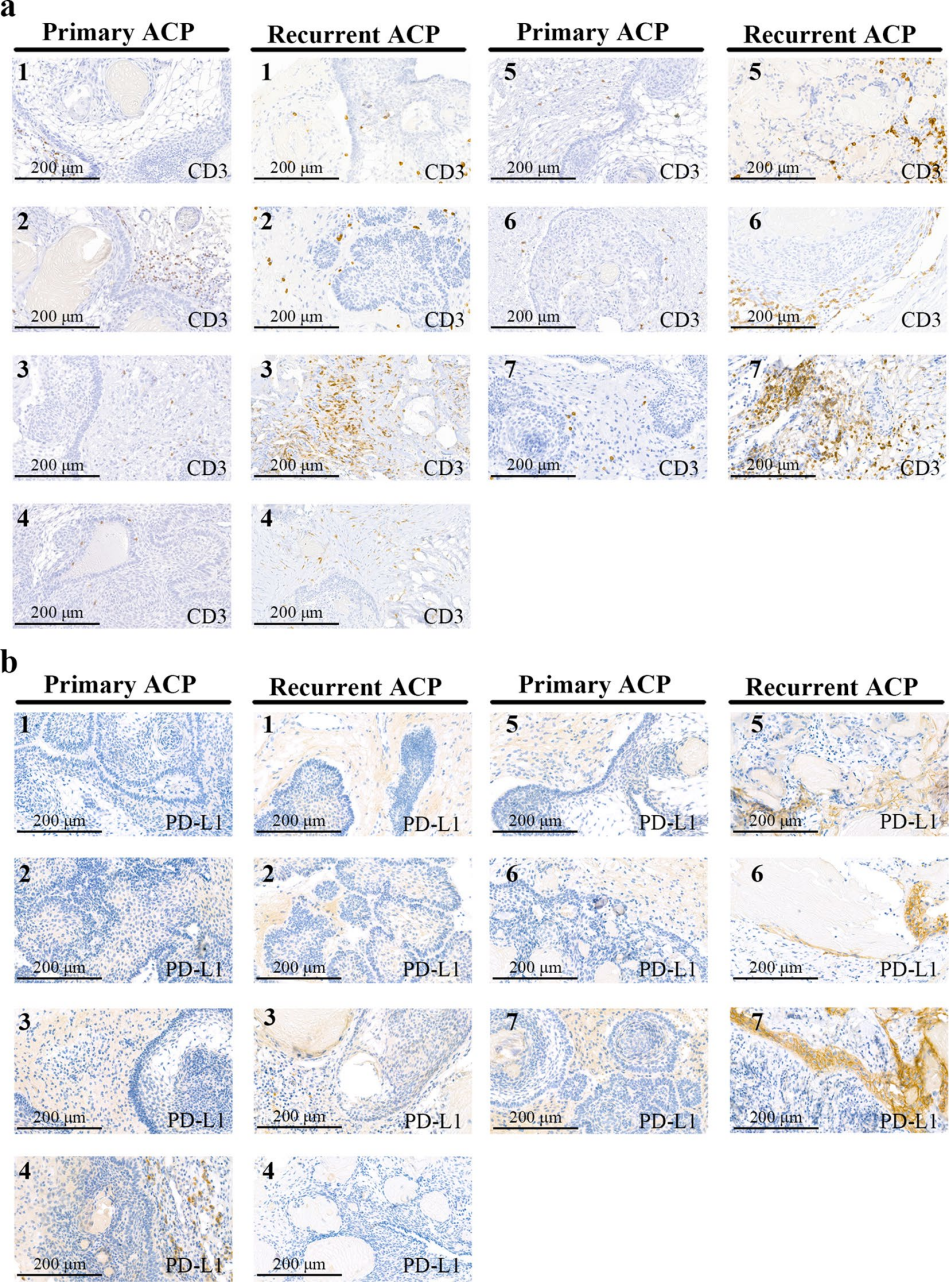
Representative immunohistochemical images of paired primary and recurrent adamantinomatous craniopharyngiomas (ACPs). (**a**) CD3 staining, (**b**) PD-L1 staining

Representative images of IHC staining for CD3 and PD-L1 are shown in Fig. 8. For the primary ACP tissues, IHC showed that most T cells were located outside the typical finger-like protrusion structure of ACP. Recurrent ACP in our cohort tended to lose the typical ACP structure comprising palisade-like epithelial cell layers and helical cell clusters. Furthermore, T cells in recurrent ACP displayed a mixed pattern (Fig. 8a). Since we showed that tumour-infiltrating T cells in ACP were partially exhausted, we investigated whether PD-L1 expression was significantly different between primary and recurrent ACPs. IHC staining results showed that PD-L1 expression was low in both conditions. Additionally, PD-L1 expression in recurrent ACP displayed a palisade-like pattern (Fig. 8b).

## Discussion

In the present study, we performed a proteome-wide bioinformatics analysis to investigate the similarities and differences between primary and recurrent ACP and validated these results experimentally.

DEPs between primary and recurrent ACPs were enriched for ECM organisation, Naba matrisome-associated functioning, collagen metabolic processes, chemotaxis, inflammatory response, and other biological processes. ECM organisation, Naba matrisome-associated activity, and collagen metabolic processes are all related to the ECM. The ECM can promote cell migration and differentiation and trigger EMT, which is important in the progression and metastasis of various tumours [18]. EMT also plays a crucial role in ACP development. ACP cells overexpress ECM-associated proteins, which in turn mediate EMT in ACP cells, promoting tumour proliferation, migration, and invasion [19].

ACP is thought to be driven by mutations in exon 3 of *CTNNB1* [18]. Previous study has shown that β-catenin may regulate the expression of *CDH1* in ACP, which is the most critical gene for the occurrence and development of ACP; moreover, its decreased expression has been associated with tumour recurrence [11]. However, Preda et al. found that CDH1 expression in ACP was unrelated to β-catenin [20]. In addition, Barros et al. observed that CDH1 expression did not correlate with tumour recurrence [18]. In our proteomic analysis, we found no difference in β-catenin expression between recurrent and primary ACPs. Furthermore, the western blotting results showed that CDH1 expression was not significantly different between the recurrent and primary tumours, whereas increased expression of CDH2 was observed in the recurrent ACP group.

We selected 24 proteins for further analysis according to the statistical results of the 12 algorithms used in Cytoscape (Table S2), including CTSK, LUM, COL14A1, and CXCL12. We found that CTSK is involved in ECM organisation, Naba matrisome-associated activity, chemotaxis, and collagen metabolic processes. CTSK is the most active mammalian collagenic enzyme [21] and is usually highly expressed in osteoclasts [22] and plays a role in mediating ECM conversion, collagen degradation, bone resorption, and ECM remodelling [23].

CTSK overexpression has also been observed in malignant tumours and heart failure [24]. This enzyme is highly expressed in the sera and tissues of patients with cancer [25]. CTSK is reportedly involved in cancer progression and invasion through TME remodelling. Furthermore, CTSK plays an important regulatory role in tumour invasion and metastasis by degrading the collagen matrix. Previous study has indicated that MMP-2 contributes to the migration and invasion of several tumours via similar mechanisms [26]. Moreover, CTSK activates pre-MMP-9 to generate MMP-9 [27] under acidic conditions, promoting tumour cell migration [28] and metastasis [29]. Several other extracellular stroma-associated proteins specifically linked to CTSK, including SPARC and TNC, are reportedly highly expressed in cancer tissue and promote the invasion and metastasis of multiple cancers [30, 31]. In addition, overexpression of bone marrow-derived CTSK may promote bone metastasis and its participation in the pathogenesis of bone tumour progression [32].

In a study of ACP in children, MMP-2 expression was higher in recurrent than in primary craniopharyngiomas [33]. Another proteomic study showed that multiple proteins, such as MMP-9, MMP-12, MAP2, TNC, and CD133, were overexpressed in ACP. Further, 16 genes, including MMP-9, CTSK, ITGB3, and FN1, were upregulated in recurrent ACP [34].

In our DEP analysis, CTSK expression in recurrent ACP was significantly higher than that in primary ACP, and elevated CTSK expression was observed in all comparisons of paired recurrent and primary ACP expression data from the same patient. MMP-2 was also highly expressed in recurrent ACP (Table S2) and included in four enriched pathways. Western blotting analysis revealed elevated CTSK and MMP-9 expression in primary ACP tissues from patients diagnosed with recurrent ACP. However, the expression of MMP-2 in recurrent tumour tissues was not significantly different from that in primary tumour tissues, suggesting that MMP-2 expression may differ individually in recurrent ACP. These results, which are consistent with those of published studies, suggest that CTSK is significantly correlated with ACP recurrence and could be a potential target for future investigation.

In the current study, we found that LUM was mainly involved in ECM organisation. Previous studies have shown that LUM is expressed in various cancer tissues and is either positively or negatively correlated with tumour progression [35], influencing cell proliferation, motility, apoptosis, autophagy, angiogenesis regulation, inflammation, immunity, and other functions [36]. Significantly increased LUM expression was observed in the DEP analysis; however, the western blotting results showed no significant difference in expression between recurrent and primary tumour tissues. This could be due to the small sample size and unpaired tumour tissues assessed in the western blotting analysis. The role of LUM in ACP recurrence requires further exploration using larger sample sizes and different age groups.

Collagen is the most abundant ECM protein; specifically, collagen VI is important [37] and abundant in tumours. The major collagen VI subtypes are encoded by three genes, COL6A1, COL6A2, and COL6A3, which are considerably upregulated in multiple cancers [38]. These three genes appeared in the ECM organisation and collagen metabolic process pathways enriched in our study and were highly expressed in recurrent ACP (Table S2). Further, COL14A1 expression was higher in recurrent than in primary ACP tissues. Few studies on COL14A1 in ACP have been conducted; therefore, the role of COL14A1 in relapse requires further investigation.

The activation of inflammatory and immune responses plays a key role in ACP pathogenesis [39]. Unlike most brain tumours, ACPs can frequently make contact with infiltrating immune cells owing to the lack of limitations of the blood–brain barrier [40–42]. Study has shown that many immune cells infiltrate and form tight adhesions between the ACP and important brain structures such as the hypothalamus. The inflammatory reaction between the tumour and these structures may lead to challenging tumour anatomy during surgery, which could cause serious postoperative complications and recurrence [43]. Tumour-infiltrating T cells have exhibited an immunosuppressive effect on the ACP TME and potentially play a role in ACP progression [44]. In our study, all three samples analysed using flow cytometry displayed high T-cell infiltration and diverse proportions of exhausted T cells, indicating a common immunosuppressive microenvironment. These results suggest that T cells in the ACP TME play an important role in ACP progression. The immune checkpoint molecules PD-1 and PD-L1 are overexpressed in ACP epithelial cells [45] and play an important role in the development of ACP [46–48]. However, the IHC staining results for PD-L1 were not significantly different between primary and recurrent ACPs.

By secreting immune-related cytokines and chemokines, immune cells can induce overactivation of intracellular signalling pathways or activation of abnormal signalling pathways, promoting tumour proliferation, invasion, and metastasis [43]. In addition, chemokines may induce the directed migration of leukocytes and participate in the inflammatory and immune responses of tumours [49]. Supporting this, Gong et al. confirmed that the high expression of various cellular chemokines in ACP was associated with poor relapse-free survival [50].

Through our analysis of disease and biological function, we observed that cellular movement of cancer and organismal injury and abnormalities were the primary pathways associated with ACP; these pathways are also involved in tumour migration, immunity, and inflammation, which is consistent with the results of our Metascape analysis.

In our study, CXCL12 was involved in the Naba matrisome-associated and chemotaxis pathways. CXCL12 is an important α-chemokine that chiefly binds to its homologous receptor CXCR4 [51]. Activation of the CXCL12/CXCR4 biological axis plays an essential role in the pathophysiology of several cancer types, including progression, recurrence, and metastasis.

CXCL12 and CXCR4 participate in antigen recognition by T and B cells and modulate the TME. CXCL12 secreted by tumour cells recruits immune cells, such as M2-type tumour-associated macrophages, T regulatory cells, and myeloid-derived suppressing cells, and guides them to produce specific immunosuppressive responses [52], which results in the promotion of tumour growth [53]. Furthermore, CXCL12 prevents naïve CD8 + T cells from differentiating into effector T cells and further into cytotoxic and memory CD8 + T cells [54]. It also interacts with CXCR4 on the surface of mature B cells, thereby mediating the recruitment of B regulatory cells to the tumour site and inhibiting T-cell activity.

Previous studies have shown that ACP recurrence and relapse-free survival are significantly correlated with CXCL12 and CXCR4 expression [34, 55]. Moreover, activation of the CXCL12/CXCR4 signalling pathway reportedly increases MMP-9 and VEGF expression in glioblastoma [56]. Studies have demonstrated that MMP-9 and VEGF play substantial roles in ACP recurrence [57]. Our analysis revealed that CXCL12 expression was higher in patients with recurrent ACP than in those with primary ACP. However, no significant difference in CXCR4 expression between recurrent and primary ACP was identified. Additionally, the western blotting results showed no significant difference in VEGF expression between recurrent and primary tumour tissues.

This study had some limitations. The sample size was small owing to the rarity of ACP in clinical practice, and the tumour tissues used for Western blotting were from unpaired primary and recurrent ACP patients. Therefore, we plan to expand this study to validate our results. We will continue to explore the specific mechanisms of action of several key proteins associated with ACP recurrence in the present study.

## Conclusion

Our research provides a deeper understanding of the proteome of the solid components of ACP and highlights several proteins associated with ACP recurrence that, to our best knowledge, have not been previously reported. The key proteins identified in this study may be used as targets for molecular therapy or immunotherapy, thus guiding the development of new and safe therapies to prevent or delay ACP recurrence.

### Electronic supplementary material

Below is the link to the electronic supplementary material.

Supplementary Material 1: Table S1

Supplementary Material 2: Fig. S1

Supplementary Material 3: Table S2

## Data Availability

The datasets used and/or analysed during the current study are available from the corresponding author on reasonable request.

## Abbreviations

ACPs: Adamantinomatous craniopharyngiomas
DEPs: Differentially expressed proteins
DIA-MS: Data-independent acquisition-mass spectrometry
DTT: Dithiothreitol
ECM: Extracellular matrix
EMT: Epithelial–mesenchymal transition
FMO: Fluorescence-minus-one
HCD: Higher-energy C-trap dissociation
HRP: Horseradish peroxidase
IAM: Iodoacetamide
IPA: Ingenuity Pathway Analysis
PBS: Phosphate-buffered saline
PPI: Protein–protein interaction
RCC: Rathke’s cleft cyst
STRING: Search Tool for the Retrieval of Interacting Genes/Proteins
TBST: 0.2% Tween-20 in Tris-buffered saline
TILs: Tumour-infiltrating lymphocytes
