# Supplementary figures and images for: Proteomics study of primary and recurrent adamantinomatous craniopharyngiomas
Source: Clin Proteomics. 2024 Apr 9;21:29. doi: 10.1186/s12014-024-09479-4 (PMC11003072; doi:10.1186/s12014-024-09479-4)

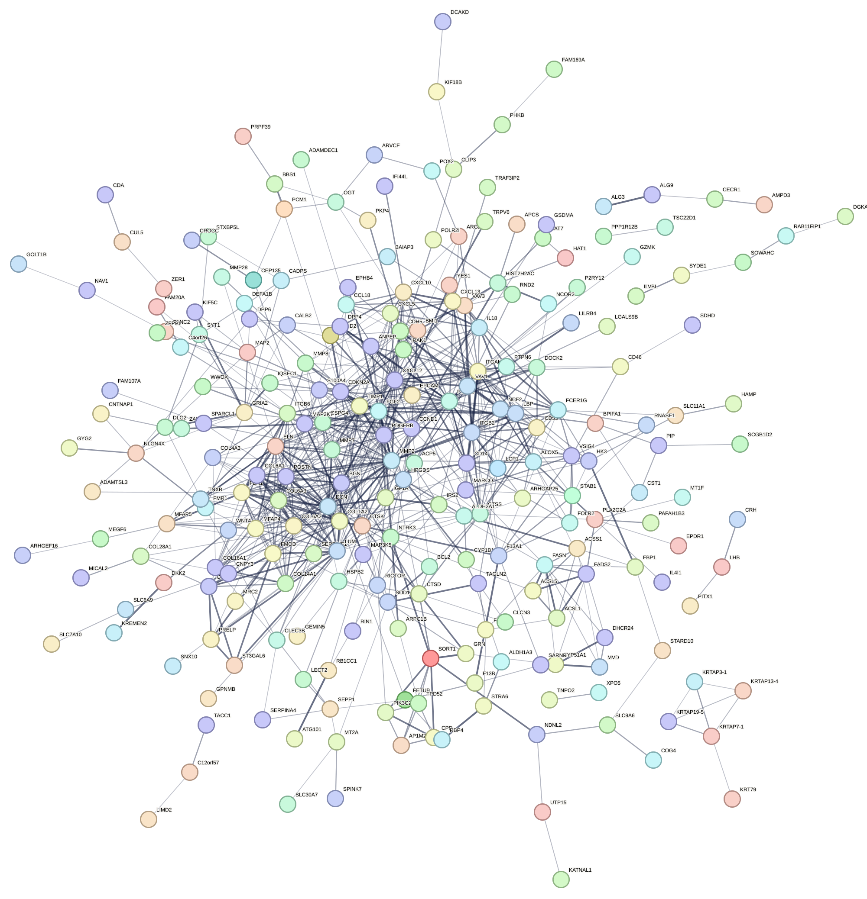

Supplement: Supplementary file 2 — Supplementary Material 2: Fig. S1 [file 12014_2024_9479_MOESM2_ESM.tif]
